# The Practice of Birth-Control

**Published:** 1921-04-30

**Authors:** 


### THE EDITOR'S LETTER-BOX.

**Correspondence on all subjects is invited, but we cannot in any way be responsible for the opinions expressed by our correspondents, who must give their name and address as a guarantee of good faith, but not necessarily for publication. Correspondents are reminded that brevity of style and conciseness of statement greatly facilitate early insertion.**

### "The Practice of Birth-Control."

*To the Editor of* The Hospital.

Sir,—"One of a Large Family," in his letter on this topic in your last issue, seems to me to present a very incomplete thesis. Leaving aside, for argument's sake, that he is generalising from one individual case, let us consider his three families of twenty-nine cousins, who have between them produced so far but four children in the next generation. Even allowing for four killed in action, and some as yet immature, this lamentable state of things surely bears out the contention of your article of the previous week that race-suicide is rampant in the middle classes: the only alternative seems to be that members of large families are in themselves likely to be sterile, which is biologically unlikely, and, in fact, is known not to be the case.

Your correspondent expresses the opinion that "very large families, in any grade of society, are a pernicious form of race-suicide," and that the reasons are far graver than mere selfishness. He would be more convincing if he told us what the reasons are. Clearly he does not mean that members of large families are economically handicapped in the race of life, because the plutocracy and the proletariat are not thus affected, only the middle classes; and he refers to all grades of society. If he adopts the argument that quality is better than quantity, where and how does he propose to draw the line? If six children in a family are likely to be of higher average quality than ten (a thing he makes no attempt to prove), will not three be argued by his readers to be better still, and one better than three? Is he not, as a matter of fact, merely playing into the hands of the selfish by giving them a handy excuse for not begetting families? Surely to spread abroad the knowledge of how to prevent conception among those classes that have it not is likely to produce the same result as has already attended the spread of this knowledge in other classes—namely, race-suicide. Which, sir, I apprehend, was the argument your article intended to enforce. The three families of cousins, of whom he speaks, are proving themselves less courageous than their four fallen war heroes: that at least is the view of yours faithfully,

One of Eight.

